# Five Glutathione S-Transferase Gene Variants in 23,452 Cases of Lung Cancer and 30,397 Controls: Meta-Analysis of 130 Studies

**DOI:** 10.1371/journal.pmed.0030091

**Published:** 2006-03-07

**Authors:** Zheng Ye, Honglin Song, Julian P. T Higgins, Paul Pharoah, John Danesh

**Affiliations:** **1**Department of Public Health and Primary Care, University of Cambridge, Strangeways Site, Cambridge, United Kingdom; **2**Department of Oncology, University of Cambridge, Strangeways Site, Cambridge, United Kingdom; **3**Public Health Genetics Unit and MRC Biostatistics Unit, Institute of Public Health, Cambridge, United Kingdom; Guy's King's and St Thomas' School of MedicineUnited Kingdom

## Abstract

**Background:**

Glutathione S-transferases (GSTs) are known to abolish or reduce the activities of intracellular enzymes that help detoxify environmental carcinogens, such as those found in tobacco smoke. It has been suggested that polymorphisms in the *GST* genes are risk factors for lung cancer, but a large number of studies have reported apparently conflicting results.

**Methods and Findings:**

Literature-based meta-analysis was supplemented by tabular data from investigators of all relevant studies of five *GST* polymorphisms ( *GSTM1* null, *GSTT1* null, I105V, and A114V polymorphisms in the *GSTP1* genes, and *GSTM3* intron 6 polymorphism) available before August, 2005, with investigation of potential sources of heterogeneity. Included in the present meta-analysis were 130 studies, involving a total of 23,452 lung cancer cases and 30,397 controls. In a combined analysis, the relative risks for lung cancer of the *GSTM1* null and *GSTT1* null polymorphisms were 1.18 (95% confidence interval [CI]: 1.14–1.23) and 1.09 (95% CI: 1.02–1.16), respectively, but in the larger studies they were only 1.04 (95% CI: 0.95–1.14) and 0.99 (95% CI: 0.86–1.11), respectively. In addition to size of study, ethnic background was a significant source of heterogeneity among studies of the *GSTM1* null genotype, with possibly weaker associations in studies of individuals of European continental ancestry. Combined analyses of studies of the 105V, 114V, and GSTM3*B variants showed no significant overall associations with lung cancer, yielding per-allele relative risks of 1.04 (95% CI: 0.99–1.09), 1.15 (95% CI: 0.95–1.39), and 1.05 (95% CI: 0.89–1.23), respectively.

**Conclusions:**

The risk of lung cancer is not strongly associated with the I105V and A114V polymorphisms in the *GSTP1* gene or with *GSTM3* intron 6 polymorphism. Given the non-significant associations in the larger studies, the relevance of the weakly positive overall associations with the *GSTM1* null and the *GSTT1* null polymorphisms is uncertain. As lung cancer has important environmental causes, understanding any genetic contribution to it in general populations will require the conduct of particularly large and comprehensive studies.

## Abstract 2

Large meta-analysis finds little evidence for a link between gene variants encoding inactive or less active variants of detoxifying enzymes and lung cancer risk.

## Introduction

Glutathione S-transferases (GSTs, Enzyme Commission 2.5.1.18) are a large family of cytosolic enzymes that catalyze the detoxification of reactive electrophilic compounds, including many environmental carcinogens (e.g., benzo[a]pyrene and other polycyclic aromatic hydrocarbons) [ 1, 2]. Inter-individual variability in GST enzyme activity is believed to confer differences in susceptibility to cancers with major environmental determinants such as lung cancer [ 3, 4, 5]. Some genetic variants in the glutathione S-transferase genes, such as the *GSTM1* null polymorphism, are known to abolish enzyme activities ( Table 1). Because individuals with the *GSTM1* null genotype have been reported to have higher levels of polycyclic aromatic hydrocarbon-dGMP adducts (which can induce genetic mutations) in lung tissue than those with the *GSTM1* genotype [ 4], such genetic variants have been extensively studied as candidates for lung cancer susceptibility, but studies have yielded apparently conflicting results [ 6– 142]. This may be due, in part, to involvement of only a few hundred cases and a few hundred controls in most studies, too few to assess reliably any moderate genetic effects in lung cancer. The interpretation of these studies has been further complicated by studies involving: (i) different *GST* polymorphisms, (ii) populations with different background smoking patterns and with different ethnic compositions (e.g., European and African populations have substantially different frequencies of certain *GST* genetic variants), and (iii) different control groups (e.g., population versus hospital based).

**Table 1:**
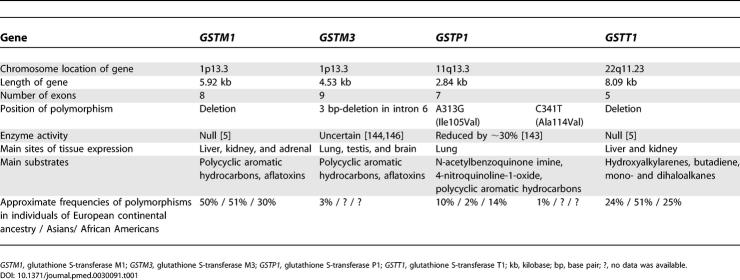
Description of Glutathione S-Transferase Polymorphisms

Five common variants in four *GST* genes ( *GSTM1, GSTT1, GSTP1,* and *GSTM3,* described in Table 1) have been studied extensively in relation to lung cancer, with each associated with completely lost or reduced activities of certain xenobiotic metabolizing enzymes: (i) the GSTM1*0 ( *GSTM1* null), (ii) the GSTT1*0 ( *GSTT1* null) alleles represent deletions of the *GSTM1* and *GSTT1* genes, respectively, with each conferring a total loss of activity in their corresponding enzymes [ 5], (iii) the A to G transition in *GSTP1* that gives rise to the Ile105Val polymorphism (also known as I105V), (iv) the C to T exchange at position 341 in the same gene, which results in the Ala114Val polymorphism (also known as A114V) (both of these *GSTP1* polymorphisms confer moderately reduced enzyme activity) [ 143], and (v) the *GSTM3* intron 6 polymorphism, a 3-base pair deletion in intron 6, which is in linkage disequilibrium with the *GSTM1* genotype and contains a recognition motif for the YY1 transcription factor, which has been postulated to regulate gene expression [ 144– 146].

The present report provides a meta-analysis of published genetic association studies, supplemented by tabular data received from study investigators of these five *GST* variants and risk of lung cancer. It includes an updated meta-analysis of 119 association studies of the *GSTM1* null polymorphism and lung cancer (involving a total of 19,729 cancer cases and 25,931 controls, about three times as many participants as in previous such reviews [ 147– 149]), as well as completely new syntheses of four other *GST* polymorphisms (i.e., the *GSTT1* null polymorphism, I105V, and A114V polymorphisms in the *GSTP1* gene, and the *GSTM3* intron 6 polymorphism). Hence, in aggregate, the present meta-analysis involves a total of 23,452 cancer cases and 30,397 controls in 130 studies (with the cases and controls in each study counted only once).

## Methods

### Data Searching

Genetic association studies published before August, 2005, investigating at least one of the five polymorphisms in the *GST* genes described above and lung cancer risk were sought by computer-based searches, scanning of the reference lists for all relevant studies and review articles (including meta-analyses), hand searching of relevant journals, and correspondence with authors of included studies. Computer searches of PubMed, Web of Science, EMBASE, and CNKI ( http://www.cnki.net) used keywords relating to the relevant genes (e.g., *“GSTT1,” “GSTP1,” “GSTM3,”* and “glutathione S-transferase”) in combination with words related to lung cancer or lung-neoplasms without language restriction. All relevant studies identified were included apart from one study in which genotype frequencies were unavailable [ 120]. Twenty-two reports involved overlapping or duplicated data with studies already included in the present review [ 121– 142].

### Data Abstraction

The following information was abstracted from each study according to a fixed protocol: study design, geographical location, ethnic group of participants, definition and numbers of cases and controls, DNA extraction and genotyping methods, frequency of genotypes, mean age of lung cancer cases, and proportion of lung cancer cases who were male ( Table S1). Previously un-reported data were included from four of these studies following correspondence that sought genotype frequencies on polymorphisms described in this review that were not reported in the original publications. In the few instances in which genotype frequencies provided by the investigators in tabular data differed slightly from published figures, the tabular data were used. Studies with different ethnic groups, and different source of controls were considered as individual studies for our analyses [ 6, 9, 10, 17, 23, 27, 41, 55].

### Statistical Methods

The per-allele odds ratio (“relative risk”) of the rare allele (105V, 114V, GSTM3*B) was compared between cases and controls by assigning scores of 0, 1, and 2 to common homozygote, heterozygote, and rare homozygote, respectively, and calculating odds ratios per unit score by logistic regression; this is analogous to modeling a co-dominant model of inheritance. Subsidiary analyses involved dominant and recessive genetic models, where possible. For *GSTM1* and *GSTT1* status with only two possible genotypes, the odds ratio was compared between cases and controls by the Mantel-Haenszel method. To make some allowance for multiple comparisons, 99% confidence intervals (CI) were used for individual studies, and 95% CI were reserved for the combined estimates. Random-effects and fixed-effect summary measures were calculated as inverse-variance weighted average of the log odds ratios. Heterogeneity was assessed using the I ^2^ statistic, which describes the proportion of variation in the log odds ratios that is attributable to genuine differences across studies rather than to random error [ 150], and by the χ ^2^ test [ 151]. The among-study variance (τ ^2^) was used to quantify the degree of heterogeneity among studies [ 152]. Percentage of τ ^2^ explained is used to describe the extent to which study-level characteristics (e.g., sample size, sources of controls) explain heterogeneity, whereas the χ ^2^ test for interaction compares meta-analyses performed within types of participants (e.g., cancer type, smoking status). Subsidiary analyses involved subgroup analyses or random-effects meta-regression. Publication bias was assessed using funnel plots (so-called because, in the absence of publication bias, such plots resemble symmetrical inverted funnels), Begg's test [ 153] and the Trim and Fill method [ 154], which estimates the number and outcomes of potentially missing studies due to publication bias. Study size ( ≥ 500 cases, 200–499 cases, and < 200 cases), source of controls (e.g., population versus hospital based), ethnicity (individuals of European continental ancestry, East Asian, and other), and cancer type (squamous, adenocarcinoma, small cell carcinoma, and other) were pre-specified as characteristics for assessment of heterogeneity; other potentially relevant subgroup analyses (such as age, sex, and smoking status) could not reliably be investigated because individual participant data were not available in this meta-analysis. Statistical analyses were done using Stata (version 8.0) statistical software (Stata Corporation, College Station, Texas, United States). Because data on the joint occurrence of genetic variants (such as the I105V and A114V polymorphisms) have generally not been reported in published studies, analyses of such haplotypes (i.e., combination of alleles at multiple positions along a genomic segment of a single chromosome) cannot be provided in this review. In the figures, areas of squares of individual studies (or sets of studies) are inversely proportional to the variances of the log odds ratios, and the horizontal lines represent CIs. Studies in the figures are listed in descending order of statistical weight.

## Results

A total of 130 relevant genetic association studies (126 published and four unpublished) were identified, with 48 studies genotyping more than one variant ( Table S1). For the *GSTM1* null polymorphism, 119 studies involved a total of 19,729 cases and 25,931 controls (weighted mean age of cases 63 years; 55% male). For the *GSTT1* null polymorphism, 44 studies involved a total of 9,636 cases and 12,322 controls (weighted mean age of cases 61 years; 59% male). For the *GSTP1* I105V polymorphism, 25 studies involved a total of 6,221 cases and 7,602 controls (weighted mean age of cases 64 years; 68% male) and for the A114V polymorphism of the same gene, four studies involved a total of 1,251 cases and 1,295 controls (weighted mean age of cases 61 years; 52% male) [ 29, 77, 92, 95]. For *GSTM3* intron 6 polymorphism, five studies involved a total of 1,238 cases and 1,179 controls (weighted mean age of cases 62 years; 85% male) [ 37, 70, 73, 78, 88].

Studies were conducted in a wide range of geographical settings, with 48% of lung cancer cases being individuals of European continental ancestry, 37% East Asian, and 15% of other ethnic origins (including African American, Turkish, and Mexican American) [ 8, 17, 22, 41, 64, 67]. Of the 130 studies, 124 were retrospective case-control studies and six were prospective in design (cohort or nested case-control) [ 32, 56, 61, 65, 78, 95]. Of the 124 retrospective studies, 80 involved controls drawn at random from approximately general populations (e.g., population registers), 25 involved controls from health-check visits or outpatient clinics, and 19 involved controls drawn from groups of patients free of cancer. Of the 124 retrospective studies, 20 matched controls to cases by age, and a further 26 matched controls to cases by age and at least one other risk factor. All but one study [ 99] used polymerase chain reaction/restriction fragment length polymorphism with various restriction enzymes to perform genotyping (the remaining study used oligonucleotide probes).

**Figure 1:**
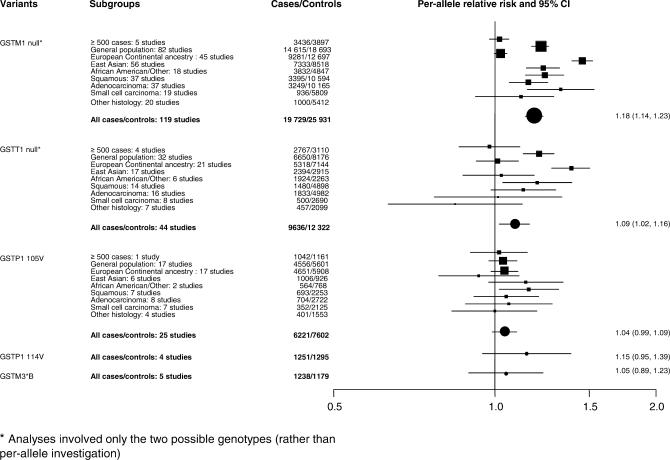
Meta-Analyses of Studies of Lung Cancer and Five *GST* Gene Polymorphisms ( *GSTM1* null, *GSTT1* null, and *GSTP1* I105V, *GSTP1* A114V, and *GSTM3* intron 6) Grouped by Various Characteristics

**Figure 2:**
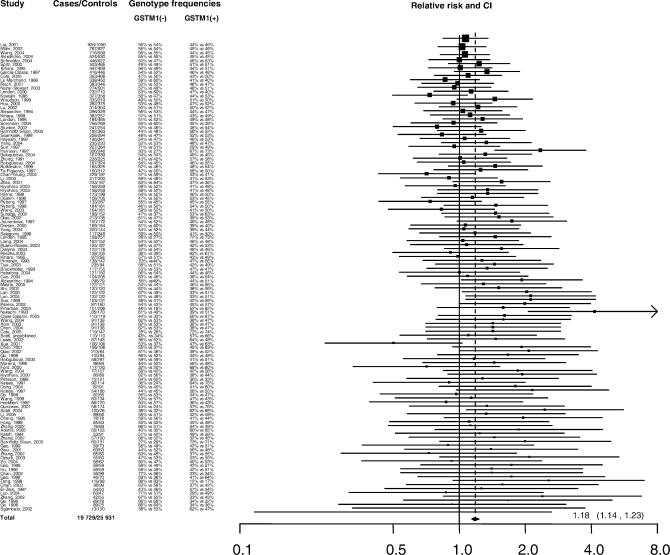
Meta-Analysis of Studies of *GSTM1* Polymorphism and Lung Cancer The horizontal axis is plotted on a log doubling scale.

There was evidence of a moderate degree of heterogeneity among the 119 studies of the *GSTM1* null genotype (I ^2^ = 44%, 95% CI: 30% to 55%, *p* < 0.0001). Study characteristics such as sample size (explaining 18% of τ ^2^, *p* < 0.0001), and ethnicity (44% of τ ^2^, *p* < 0.0001) explained much of the heterogeneity, whereas source of controls (0% of τ ^2^, *p* = 0.82) and cancer type (χ ^2^_3_ = 1.49, *p* = 0.69) explained a relatively small fraction. Overall, the relative risk for lung cancer risk of the *GSTM1* null genotype was 1.22 (95% CI: 1.16–1.30) using a random-effects model and 1.18 (95% CI: 1.14–1.23; Figures 1 and 2) using a fixed-effect model, but a funnel plot of these 119 studies suggested a possibility of the preferential publication of strikingly positive findings in smaller studies (Begg's test, *p* < 0.0001; Figure S1). Analysis restricted to the five studies with at least 500 cases (total of 3,436 cases and 3,897 controls), which should be less prone to selective publication than are the smaller studies, yielded a relative risk of 1.04 (95% CI: 0.95–1.14), which is not statistically significant. Further evidence of selective publication derives from the results of the Trim and Fill approach, which suggested that 28 missing studies are required to make the funnel plot symmetrical ( Figure S2).

**Figure 3:**
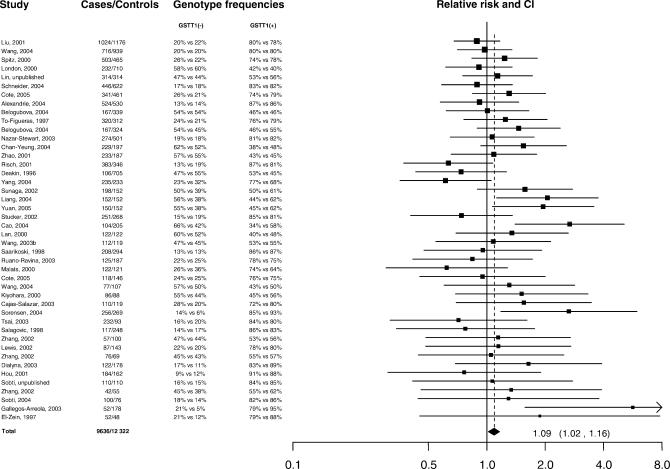
Meta-Analysis of Studies of *GSTT1* Polymorphism and Lung Cancer The horizontal axis is plotted on a log doubling scale.

There was a moderately high degree of heterogeneity among the 44 studies of the *GSTT1* null genotype (I ^2^ = 57%, 95% CI: 40% to 70%, *p* < 0.0001). Study characteristics such as sample size (17% of τ ^2^ explained, *p* = 0.01) and ethnicity (31% of τ ^2^, *p* = 0.02) explained some of the heterogeneity, but source of controls (0% of τ ^2^, *p* = 0.02) and cancer type (χ ^2^_3_ = 5.55, *p* = 0.14) did not explain much of it. Overall, the relative risk for lung cancer of the *GSTT1* null genotype was 1.13 (95% CI: 1.02–1.26) using a random-effects model and 1.09 (95% CI: 1.02–1.16; Figures 1 and 3) using a fixed-effect model. A funnel plot did not indicate the presence of publication bias in these studies (Begg's test *p* = 0.14; plot available on request), and an analysis of the four studies with at least 500 cases (total of 2,767 cases and 3,110 controls) yielded a relative risk of 0.99 (95% CI: 0.86–1.11).

**Figure 4:**
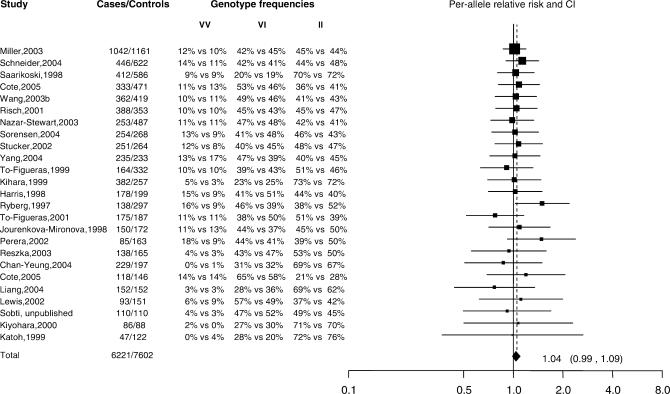
Meta-Analysis of Studies of *GSTP1* I105V Polymorphism and Lung Cancer The horizontal axis is plotted on a log doubling scale.

**Figure 5:**
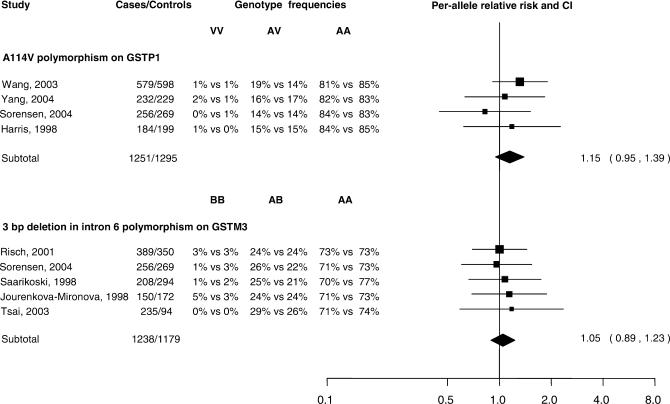
Meta-Analysis of Studies of *GSTP1* A114V and *GSTM3* Intron 6 Polymorphisms and Lung Cancer The horizontal axis is plotted on a log doubling scale.

There was no significant heterogeneity among the 25 studies of the 105V variant in the *GSTP1* gene and lung cancer (I ^2^ = 0% 95% CI: 0% to 44%, *p* = 0.66). Overall, the per-allele relative risk for lung cancer of the 105V variant was 1.04 (95% CI: 0.99–1.09; Figures 1 and 4), with corresponding results under dominant and recessive genetic models of 1.02 (95% CI: 0.95–1.09) and 1.13 (95% CI: 1.01–1.27), respectively. There was no significant heterogeneity among the four studies of the 114V variant of the same gene (I ^2^ = 0%, 95% CI: 0% to 85%, *p* = 0.39). The overall per-allele risk of the 114V variant for lung cancer was 1.15 (95% CI: 0.95–1.39), with corresponding results under dominant and recessive genetic models of 1.17 (95% CI: 0.95–1.45) and 1.27 (95% CI: 0.48–3.33; Figures 1 and 5), respectively. There was no significant heterogeneity among the five studies of the GSTM3*B variant (I ^2^ = 0%, 95% CI: 0% to 79%, *p* = 0.95). The overall per-allele risk of the GSTM3*B variant for lung cancer was 1.05 (95% CI: 0.89–1.23; Figures 1 and 6), with corresponding results under dominant and recessive genetic models of 1.11 (95% CI: 0.92–1.34) and 0.91 (95% CI: 0.52–1.57), respectively.

## Discussion

The present meta-analysis of 130 genetic association studies involves more than 23,000 cases and 30,000 controls and provides the most comprehensive assessment so far of the relevance to lung cancer of five *GST* gene polymorphisms. This review indicates that the risk of lung cancer is not strongly associated with the I105V and A114V polymorphisms in the *GSTP1* gene or with the *GSTM3* intron 6 polymorphism. The relevance of the weakly positive overall associations observed of the *GSTM1* null and of the *GSTT1* null genotypes with the risk of lung cancer is uncertain, particularly given the non-significant relative risks observed for each variant in the larger studies (which should be less prone to selective reporting than are the smaller studies). At least for the studies of the *GSTM1* null variant, there is a possibility that the magnitude of the association varies significantly by characteristics such as ethnic background.

The potential limitations of the present report merit consideration. Although the available data on three of the polymorphisms (i.e., *GSTM1* null, *GSTT1* null, and *GSTP1* 105V) comprise at least 6,000 cases and at least 7,000 controls for each of these variants, the present data on the *GSTP1* 114V and the GSTM3*B variants (comprising only about 1,000 cases and about 1,000 controls for each) are much sparser—too few to enable reliable assessments of any per-allele relative risks of about 10% to 20%. Even for the more extensively studied variants, moreover, the present data are insufficient to enable reliable assessment of the impact of the genotypes in potentially relevant subgroups, such as those defined by ethnic background or by cancer histology. Lack of individual data in the present review prevents more detailed analyses, such as any joint effects of gene-gene or gene-environment factors. Funnel plots suggest publication bias in studies of only one of the five variants considered (i.e., the *GSTM1* null genotype), but it is difficult to exclude this bias in studies of the four other variants despite our attempts to identify un-reported data by correspondence with investigators and exploration of data for the possible effects of selective publication.

The pathways of carcinogen metabolism are complex, mediated by the activities of multiple genes (such as *GSTM1,* and *CYP1A1* [ 3, 155]). The effect of any single gene might have more limited impact on lung cancer than has so far been anticipated. The failure to demonstrate important associations between each of the five *GST* polymorphisms and lung cancer does not necessarily exclude the possibility that other variants (or combinations of alleles at multiple positions) in the same genes could be materially relevant to lung cancer. A more comprehensive empirical approach, in which all common variants are identified by resequencing followed by the genotyping of a tagging set of variants, may prove productive [ 156, 157]. The examples in this review, therefore, illustrate the need for much larger and more comprehensive studies than have been customary in order to evaluate reliably any moderate genetic effects that might be realistically expected in a complex disease such as lung cancer, which has known major environmental determinants [ 158].

## Supporting Information

Figure S1 Funnel Plot of Studies of *GSTM1* Null Polymorphism and Lung Cancer Showing a Possible Excess of Smaller Studies with Strikingly Positive Findings beyond the 95% CI; Begg's Test, *p* < 0.0001
(52 KB PPT).

Figure S2 Trimmed and Filled Funnel Plot of *GSTM1* Null Polymorphism and Lung Cancer
The hollow diamonds are the actual studies included in the meta-analysis, the squares are the Trimmed and Filled studies required to achieve symmetry.
(56 KB PPT).

Table S1 Characteristics of Genetic Association Studies of Five *GST* Polymorphisms in the Present Meta-Analyses
(670 KB DOC).

### Accession Numbers

The UniGene ( http://www.ncbi.nlm.nih.gov/entrez/query.fcgi?db=unigene) accession numbers used in this paper are *GSTM1* (Hs.301961), *GSTM3* (Hs.2006), *GSTP1* (Hs.523836), and *GSTT1* (Hs.77490).

The GeneCard ( http://www.genecards.org) protein coding ID numbers are GSTM1 (GC01P109942), GSTM3 (GC019988), GSTP1 (GC11P067107), and GSTT1 (GC22M022700).

**Patient Summary**

**Background**

Genes and the environment determine a person's risk of cancer. For some cancers, strong environmental risk factors have been identified. One such example is lung cancer, where the large majority of cases are caused by smoking. However, some people who never smoke get lung cancer, and some heavy smokers do not. To help understand such cases, scientists have studied a group of genes called glutathione S-transferase genes. These genes contain the genetic information to make a group of proteins, the glutathione s-transferases, which detoxify environmental poisons such as those contained in cigarette smoke. Different people have slightly different versions of these genes. Some of these gene variants are thought to result in less active or even completely inactive proteins. Scientists have wondered whether these different gene variants influence the lung cancer risk in smokers and non-smokers. To find out, they have done a lot of studies, some small, some large, to test whether there are associations between particular glutathione s-transferase gene variants and the risk of lung cancer.

**Why Was This Study Done?**

Such association studies compare a group of people with lung cancer and a very similar group without lung cancer. Researchers determine the genetic make-up of both groups and ask whether a particular gene variant is more common among either the cancer patients (the “cases”) or the people without cancer (the “controls”). A variant that is more common among the cases might convey a risk, and one that is more common among the controls might convey some level of protection. The larger the studies are, and the more similar the cases and controls are, the better the chance to detect a “real” association. However, association studies are notoriously difficult to interpret, and most scientists agree that several independent studies are necessary before one can be reasonably sure that a particular gene variant conveys a risk or a protection. A rigorous way to summarize and integrate the results from several individual association studies is to do what is called a meta-analysis.

**What Did the Researchers Do and What Did They Find?**

These researchers did such a meta-analysis of 130 studies. Together, the studies tested possible associations between five relatively common variants in four glutathione s-transferase genes and the occurrence of lung cancer. All of the variants tested resulted in either less active or inactive versions of the proteins. Some of the individual studies had found associations between one or several of the variants and lung cancer (suggesting that they conveyed a risk), others had not. Altogether, the studies included data from over 23,000 lung cancer patients and 30,000 control individuals without lung cancer. After summarizing all of the results, it became clear that none of the five variants, not even the ones that resulted in inactive proteins, conveyed a clearly strong risk for lung cancer.

**What Does This Mean?**

A rigorous assessment of the results to date suggests that none of the five variants conveys a clearly strong risk for lung cancer in the general population. The gene variants included were some of the obvious ones to study, but there might be others that have a strong influence on an individual's risk to get lung cancer. It is also possible that some of these gene variants convey a stronger risk in some subgroups, for example, in particular ethnic groups that share a common genetic background. Additional studies that look specifically at the risk in particular subgroups would be needed to find out whether this is indeed the case. And much larger studies would be needed to determine reliably whether there are genes that convey a small or moderate risk for (or protection against) lung cancer.

**Where Can I Find More Information Online?**

The following pages provide information on lung cancer and cancer genetics.

Pages from the US National Cancer Institute:

http://www.cancer.gov/cancertopics/types/lung

http://www.cancer.gov/cancertopics/prevention-genetics-causes/genetics

MedlinePlus pages:

http://www.nlm.nih.gov/medlineplus/lungcancer.html

Cancer Research UK pages:

http://www.cancerhelp.org.uk/help/default.asp?page=2787

CancerIndex:

http://www.cancerindex.org/clinks2l.htm

## Abbreviations

CI: confidence interval
GST: glutathione S-transferase
