# Supplementary material for: Five Glutathione S-Transferase Gene Variants in 23,452 Cases of Lung Cancer and 30,397 Controls: Meta-Analysis of 130 Studies
Source: PLoS Med. 2006 Mar 7;3(4):e91. doi: 10.1371/journal.pmed.0030091 (PMC1391981; doi:10.1371/journal.pmed.0030091)
Supplement: Supplementary file 1 [file pmed.0030091.st001.doc]

| **Web-table: Characteristics of genetic association studies of five GST polymorphisms in the present meta-analyses.** | | | | | | | | | | |  |  |  |  |
| --- | --- | --- | --- | --- | --- | --- | --- | --- | --- | --- | --- | --- | --- | --- |
|  |  |  |  |  |  |  |  |  |  |  |  |  |  |  |
| **author** | **year** | **genotype** | **Location** | **Region** | **Ethnic** | **Control source** | **Total cases** | **Total controls** | **Study design** | **Control info** | **Control matching** | **Assay** | **% male in cases** | **mean age (years) in cases** |
|  |  |  |  |  |  |  |  |  |  |  |  |  |  |  |
| Zhong | 1991 | GSTM1 | UK | Europe | Caucasian | hospital | 228 | 225 | case control | hospital, healthy | NA | probe | NS | NS |
| Heckbert | 1992 | GSTM1 | USA | North America | Caucasian | population | 66 | 120 | case control | random selected | NA | RFLP | NS | NS |
| Hayashi | 1992 | GSTM1 | Japan | Asia | East Asia | population | 186 | 341 | case cohort | random selected | NA | PCR | NS | NS |
| Brockmoller | 1993 | GSTM1 | Germany | Europe | Caucasian | hospital | 117 | 155 | case control | hospital collection | NA | PCR | 76.1% | 69 |
| Hirvonen | 1993 | GSTM1 | France | Europe | Caucasian | population | 138 | 142 | case control | healthy | NA | PCR | 83.0% | 65 |
| Nakachi | 1993 | GSTM1 | Japan | Asia | East Asia | population | 85 | 170 | nest case control | general population | NA | PCR | NS | NS |
| Alexandrie | 1994 | GSTM1 | Sweden | Europe | Caucasian | population | 296 | 329 | case control | healthy controls | NA | PCR | 70.9% | 67.5 |
| Alexandrie | 1994 | GSTM1 | Sweden | Europe | Caucasian | hospital | 296 | 79 | case control | COPD controls | NA | PCR | 70.9% | 67.5 |
| Katoh | 1994 | GSTM1 | Japan | Asia | East Asia | population | 53 | 91 | case control | healthy | NA | PCR | NS | NS |
| Kihara | 1995 | GSTM1 | Japan | Asia | East Asia | population | 447 | 469 | case control | healthy | NA | PCR | NS | NS |
| Kihara | 1995 | GSTM1 | Japan | Asia | East Asia | population | 97 | 258 | case control | health check up | NA | PCR | 85.5% | NS |
| Kawajiri | 1995 | GSTM1 | Japan | Asia | East Asia | population | 327 | 358 | case control | healthy | NA | PCR | NS | NS |
| Cheng | 1995 | GSTM1 | USA | North America | NA | population | 78 | 78 | case control | friends or spouses of cases | NA | PCR | 56.0% | 66 |
| London | 1995 | GSTM1 | USA | North America | Caucasian | population | 184 | 465 | case control | random selected | age | PCR | 58.0% | 64 |
| London | 1995 | GSTM1 | USA | North America | African American | population | 158 | 251 | case control | random selected | age | PCR | NS | NS |
| Ge | 1996 | GSTM1 | Hong Kong | Asia | East Asia | population | 89 | 25 | case control | healthy | NA | PCR | NS | NS |
| Ge | 1996 | GSTM1 | Hong Kong | Asia | East Asia | hospital | 89 | 28 | case control | bronchiectasis patients | NA | PCR | NS | NS |
| Moreira | 1996 | GSTM1 | Portugal | Europe | Caucasian | population | 98 | 84 | case control | blood donor | NA | PCR | NS | NS |
| Deakin | 1996 | GSTM1 | UK | Europe | Caucasian | hospital | 106 | 705 | case control | lung diseases | NA | PCR | 67.0% | 62 |
| Harrison | 1997 | GSTM1 | UK | Europe | Caucasian | population | 306 | 246 | case control | blood donor | NA | PCR | NS | NS |
| Kelsey; MA | 1997 | GSTM1 | USA | North America | Mexican-Americans | population | 92 | 114 | case control | general population | age/sex/ethnicity | PCR | NS | NS |
| Kelsey; AA | 1997 | GSTM1 | USA | North America | African American | population | 54 | 186 | case control | general population | age/sex/ethnicity | PCR | NS | NS |
| Sun | 1997 | GSTM1 | China | Asia | East Asia | hospital | 207 | 364 | case control | outpatients | NA | PCR | NS | NS |
| Jourenkova | 1997 | GSTM1 | France | Europe | Caucasian | hospital | 150 | 172 | case control | cancer free | age | PCR | 93.0% | 58.4 |
| To-Figueras | 1997 | GSTM1 | Spain | Europe | Caucasian | population | 160 | 312 | case control | healthy smokers+ general population | NA | PCR | NS | NS |
| El-Zein | 1997 | GSTM1 | USA | North America | NA | population | 54 | 50 | case control | healthy | NA | PCR | NS | NS |
| Garcia-Closas | 1997 | GSTM1 | USA | North America | Caucasian | population | 416 | 446 | case control | healthy | NA | PCR | NS | NS |
| Ryberg | 1997 | GSTM1 | Norway | Europe | Caucasian | population | 133 | 287 | case control | healthy male | NA | PCR | 100.0% | 62.3 |
| Saarikoski | 1998 | GSTM1 | Finland | Europe | Caucasian | population | 208 | 294 | case control | working-aged blood donors | NA | PCR | 79.8% | 62 |
| Salagovic | 1998 | GSTM1 | Slovakia | Europe | Caucasian | population | 117 | 248 | case control | healthy | NO | PCR | 78.6% | NS |
| Tang | 1998 | GSTM1 | USA | North America | mixed | hospital | 119 | 98 | case control | inpatient | NO | PCR | 56.7% | 67 |
| Nyberg | 1998 | GSTM1 | Sweden | Europe | Caucasian | population | 184 | 161 | case control | general population | age/sex/hosptal | PCR | 25.4% | 65.1 |
| Hong | 1998 | GSTM1 | Korea | Asia | East Asia | hospital | 85 | 63 | case control | hospital collection | NA | PCR | NS | NS |
| Harris | 1998 | GSTM1 | Australia | Australia | Caucasian | population | 173 | 199 | case control | general population | NA | PCR | NS | 66 |
| Le Marchand | 1998 | GSTM1 | USA | North America | mixed | population | 338 | 450 | case control | random selected | sex/age/ethnicity | PCR | 61.0% | 64.5 |
| Gao | 1998 | GSTM1 | China | Asia | East Asia | population | 46 | 70 | case control | population based | age/sex | PCR | NS | NS |
| Hu | 1998 | GSTM1 | China | Asia | East Asia | hospital | 59 | 59 | case control | outpatients | age/sex | PCR | 72.9% | NS |
| Qu | 1998 | GSTM1 | China | Asia | East Asia | population | 100 | 94 | case control | healthy | NA | PCR | NS | 60.2 |
| Qu | 1998 | GSTM1 | China | Asia | East Asia | population | 82 | 85 | case control | healthy | NA | PCR | NS | 47.99 |
| Gao | 1999 | GSTM1 | China | Asia | East Asia | population | 59 | 73 | case control | general population | age/sex | PCR | 64.4% | 54 |
| Gao | 1999 | GSTM1 | China | Asia | East Asia | hospital | 59 | 59 | case control | outpatients | age/sex | PCR | 72.9% | 56 |
| Butkiewicz | 1999 | GSTM1 | Poland | Europe | Caucasian | population | 165 | 325 | case control | healthy | NA | PCR | NS | NS |
| Wang | 1999 | GSTM1 | China | Asia | East Asia | hospital | 60 | 134 | case control | inpatient | NA | PCR | NS | NS |
| Woodson | 1999 | GSTM1 | Finland | Europe | Caucasian | population | 333 | 319 | nested case control | healthy | age/group/data of blood draw | PCR | 100.0% | NS |
| Persson | 1999 | GSTM1 | China | Asia | East Asia | population | 75 | 121 | case control | healthy | NA | PCR | 55.3% | NS |
| Kihara | 1999 | GSTM1 | Japan | Asia | East Asia | population | 382 | 257 | case control | healthy | NA | PCR | NS | NS |
| Xue | 1999 | GSTM1 | China | Asia | East Asia | population | 103 | 131 | case control | healthy | NA | PCR | NS | NS |
| Malats | 2000 | GSTM1 | Europe | Europe | Caucasian | hospital | 122 | 121 | case control | cancer free | NA | PCR | 13.9% | NS |
| Dresler | 2000 | GSTM1 | USA | North America | Caucasian | population | 169 | 164 | case control | healthy | NA | PCR | 52.0% | 65 |
| Hou | 2000 | GSTM1 | Norway | Europe | Caucasian | population | 282 | 375 | case control | healthy | NO | PCR | NS | NS |
| Ford | 2000 | GSTM1 | USA | North America | African American | hospital | 117 | 120 | case control | hospital patients | NA | PCR | 75.2% | 59.6 |
| Lan | 2000 | GSTM1 | China | Asia | East Asia | population | 122 | 122 | case control | healthy | age | PCR | 64.8% | NS |
| Belogubova | 2000 | GSTM1 | Russia | Europe | Caucasian | population | 58 | 297 | case control | healthy donors | NO | PCR | NS | 79 |
| Spitz | 2000 | GSTM1 | USA | North America | Caucasian | population | 503 | 465 | case control | general population | age/sex/ethnicity/smoking | PCR | 52.5% | 62.3 |
| London | 2000 | GSTM1 | China | Asia | East Asia | population | 232 | 710 | cohort | general population | age/location | PCR | NS | 58.8 |
| Kiyohara | 2000 | GSTM1 | Japan | Asia | East Asia | population | 86 | 88 | case control | healthy male | NO | PCR | NS | NS |
| Zhao | 2001 | GSTM1 | Singapore | Chinese | East Asia | hospital | 233 | 187 | case control | inpatient | age | PCR | 41.2% | 65.5 |
| Risch | 2001 | GSTM1 | Germany | Europe | Caucasian | hospital | 383 | 346 | case control | inpatient | NO | PCR | 75.8% | 60.9 |
| Liu | 2001 | GSTM1 | USA | North America | NA | population | 926 | 1036 | case control | friends of cases | NO | PCR | 54.0% | 65 |
| Gsur | 2001 | GSTM1 | Austria | Europe | Caucasian | hospital | 63 | 63 | case control | cancer free | age | PCR | NS | 63.9 |
| Quinones | 2001 | GSTM1 | Chile | other | Chile | population | 58 | 174 | case control | healthy | NO | PCR | 50.6% | 50.3 |
| Chen | 2001 | GSTM1 | China | Asia | East Asia | population | 106 | 106 | case control | healthy | age/gender | PCR | 82.1% | 56.9 |
| Xue | 2001 | GSTM1 | China | Asia | East Asia | population | 106 | 106 | case control | healthy | age | PCR | NS | 56.9 |
| Sunaga | 2002 | GSTM1 | Japan | Asia | East Asia | hospital | 198 | 152 | case control | cancer free | NA | PCR | NS | NS |
| Stucker | 2002 | GSTM1 | France | Europe | Caucasian | hospital | 247 | 254 | case control | from different disorders except cancers | age/hospital | PCR | NS | 59.3 |
| Perera | 2002 | GSTM1 | USA | North America | Caucasian | population | 87 | 160 | nest case control | general population | age/smoking | PCR | 100.0% | NS |
| Miller | 2002 | GSTM1 | USA | North America | unknown | population | 767 | 927 | case control | general population | NO | PCR | 55.0% | 67 |
| Lu | 2002 | GSTM1 | China | Asia | East Asia | population | 314 | 304 | case control | healthy | age/sex | PCR | 69.7% | 58.6 |
| Lewis | 2002 | GSTM1 | UK | Europe | Caucasian | hospital | 87 | 143 | case control | patients, free of benign and malignant | NO | PCR | NS | NS |
| Sgambato | 2002 | GSTM1 | Italy | Europe | Caucasian | population | 13 | 100 | case control | inpatients and outpatients | NO | PCR | 59.1% | 62.7 |
| Zhang | 2002 | GSTM1 | China | Asia | East Asia | hospital | 65 | 60 | case control | inpatients | NA | PCR | 90.8% | 59.4 |
| Shi | 2002 | GSTM1 | China | Asia | East Asia | population | 120 | 120 | case control | healthy | age/sex | PCR | 56.7% | 57.2 |
| Qiao | 2002 | GSTM1 | China | Asia | East Asia | hospital | 213 | 64 | case control | inpatients | NA | PCR | NS | NS |
| Qiao | 2002 | GSTM1 | China | Asia | East Asia | population | 213 | 135 | case control | healthy | NA | PCR | NS | NS |
| Zhang | 2002 | GSTM1 | China | Asia | East Asia | population | 57 | 100 | case control | healthy, no smokers | NA | PCR | NS | NS |
| Zhang | 2002 | GSTM1 | China | Asia | East Asia | population | 76 | 69 | case control | healthy, >60 years | NA | PCR | NS | NS |
| Zhang | 2002 | GSTM1 | China | Asia | East Asia | population | 42 | 55 | case control | healthy, women | NA | PCR | NS | NS |
| Chan | 2002 | GSTM1 | China | Asia | East Asia | population | 56 | 99 | case control | healthy | NA | PCR | NS | NS |
| Kiyohara | 2003 | GSTM1 | Japan | Asia | East Asia | hospital | 158 | 259 | case control | hospital control | age/hospital | PCR | NS | NS |
| Tsai | 2003 | GSTM1 | USA | North America | NA | population | 235 | 94 | case control | healthy | NO | PCR | 50.0% | NS |
| Pinarbasi | 2003 | GSTM1 | Turkey | Europe | Turkish | population | 101 | 206 | case control | healthy | NO | PCR | 79.0% | 61.9 |
| Nazar-Stewart | 2003 | GSTM1 | USA | North America | Caucasian | population | 274 | 501 | case control | random digit dialing | age | PCR | NS | NS |
| Wang | 2003 | GSTM1 | China | Asia | East Asia | population | 164 | 181 | case control | healthy | age/sex | PCR | NS | NS |
| Dialyna | 2003 | GSTM1 | Greece | Europe | Caucasian | population | 122 | 178 | case control | healthy | NA | PCR | NS | NS |
| Reszka | 2003 | GSTM1 | Poland | Europe | Caucasian | hospital | 138 | 165 | case control | non-malignant patient | age/gender | PCR | 76.8% | 59.7 |
| Kiyohara | 2003 | GSTM1 | Japan | Asia | East Asia | hospital | 158 | 259 | case control | hospital non smokers | age/hospital | PCR | NS | NS |
| Cajas-Salazar | 2003 | GSTM1 | USA | North America | Caucasian | population | 110 | 119 | case control | healthy smokers | age/gender | PCR | 57.0% | 59.9 |
| Ruano-Ravina | 2003 | GSTM1 | Spain | Europe | Caucasian | hospital | 125 | 187 | case control | outpatients | age | PCR | 89.4% | 64.2 |
| Ozturk | 2003 | GSTM1 | Turkey | Europe | Turkish | population | 55 | 60 | case control | healthy | age | PCR | 85.5% | 57.8 |
| Chen | 2003 | GSTM1 | China | Asia | East Asia | population | 38 | 99 | case control | healthy | NA | PCR | 71.1% | 55.6 |
| Xian | 2003 | GSTM1 | China | Asia | East Asia | hospital | 91 | 138 | case control | outpatients, and random population | age | PCR | NS | NS |
| Schneider | 2004 | GSTM1 | Germany | Europe | unknown | hospital | 446 | 622 | case control | outpatients | age | PCR | 90.6% | 64.4 |
| Alexandrie | 2004 | GSTM1 | Sweden | Europe | Caucasian | population | 524 | 530 | case control | lab workers | NO | PCR | 34.4% | 66 |
| Yang P | 2004 | GSTM1 | USA | North America | Caucasian | population | 235 | 233 | case control | resident | NA | PCR | NS | NS |
| Chan-Yeung | 2004 | GSTM1 | China | Asia | East Asia | population | 229 | 197 | case control | healthy | NO | PCR | 67.2% | 53.8 |
| Yang XR | 2004 | GSTM1 | China | Asia | East Asia | population | 200 | 144 | case control | population based, women | age | PCR | 5.5% | NS |
| Lan | 2004 | GSTM1 | China | Asia | East Asia | population | 122 | 122 | case control | healthy | NA | PCR | 64.8% | 55.1 |
| Belogubova | 2004 | GSTM1 | Russia | Europe | Caucasian | population | 167 | 339 | case control | health blood donors | NO | PCR | 89.0% | 60 |
| Belogubova | 2004 | GSTM1 | Russia | Europe | Caucasian | hospital | 167 | 324 | case control | old outpatients | NO | PCR | 89.0% | 60 |
| Sorensen | 2004 | GSTM1 | Denmark | Europe | Caucasian | population | 256 | 269 | case cohort | healthy | age/gender/smoking | PCR | NS | NS |
| Sobti | 2004 | GSTM1 | India | Asia | East Asia | population | 100 | 76 | case control | general population | age | PCR | 95.0% | 55.5 |
| Habalova | 2004 | GSTM1 | Slovakia | Europe | Caucasian | population | 121 | 150 | case control | healthy blood donors | NO | PCR | 85.1% | 61.5 |
| Liang | 2004 | GSTM1 | China | Asia | East Asia | hospital | 152 | 152 | case control | inpatient | age/gender | PCR | 70.4% | 60.5 |
| Wang | 2004 | GSTM1 | China | Asia | East Asia | hospital | 91 | 138 | case control | outpatients | age | PCR | 65.9% | 63.2 |
| Wang | 2004 | GSTM1 | USA | North America | Caucasian | hospital | 716 | 939 | case control | outpatients | NO | PCR | 53.9% | 64.1 |
| Li | 2004 | GSTM1 | China | Asia | East Asia | population | 217 | 200 | case control | healthy | NA | PCR | NS | NS |
| Ye | 2004 | GSTM1 | China | Asia | East Asia | hospital | 58 | 62 | case control | inpatients | NA | PCR | 65.5% | NS |
| Chen | 2004 | GSTM1 | China | Asia | East Asia | hospital | 91 | 138 | case control | outpatients | age/sex | PCR | 65.9% | NS |
| Dong | 2004 | GSTM1 | China | Asia | East Asia | hospital | 82 | 91 | case control | inpatients | age/sex | PCR | NS | NS |
| Luo | 2004 | GSTM1 | China | Asia | East Asia | hospital | 63 | 47 | case control | outpatients | age/sex | PCR | 77.8% | 56.5 |
| Wang | 2004 | GSTM1 | China | Asia | East Asia | population | 77 | 107 | case control | healthy | age/sex | PCR | NS | NS |
| Cao | 2004 | GSTM1 | China | Asia | East Asia | population | 104 | 205 | case control | healthy | age | PCR | 59.0% | 57.4 |
| Cote | 2005 | GSTM1 | USA | North America | Caucasian | population | 353 | 468 | case control | random dial | age | PCR | 53.0% | 42.4 |
| Cote | 2005 | GSTM1 | USA | North America | African American | population | 119 | 147 | case control | random dial | age | PCR | 42.7% | 41.4 |
| Barnholtz-Sloan | 2005 | GSTM1 | USA | North America | Caucasian | population | 192 | 363 | case control | population based | NA | PCR | NS | NS |
| Barnholtz-Sloan | 2005 | GSTM1 | USA | North America | African American | population | 60 | 131 | case control | population based | NA | PCR | NS | NS |
| Adonis | 2005 | GSTM1 | Chile | other | Chile | population | 55 | 103 | case control | healthy | NA | PCR | NS | NS |
| Li | 2005 | GSTM1 | China | Asia | East Asia | hospital | 99 | 66 | case control | inpatients | NA | PCR | 74.7% | 58 |
| Sobti | unpublished | GSTM1 | India | Asia | East Asia | population | 110 | 110 | case control | population based | NA | PCR | NS | NS |
| Salagovic | 1998 | GSTT1 | Slovakia | Europe | Caucasian | population | 117 | 248 | case control | healthy | NO | RFLP | 78.6% | NS |
| Lan | 2000 | GSTT1 | China | Asia | East Asia | population | 122 | 122 | case control | healthy | age | RFLP | NS | NS |
| Malats | 2000 | GSTT1 | Europe | Europe | Caucasian | hospital | 122 | 121 | case control | NA | NA | RFLP | 13.9% | NS |
| Spitz | 2000 | GSTT1 | USA | North America | Caucasian | population | 503 | 465 | case control | general population | age/sex/ethnicity/smoking | RFLP | NS | NS |
| London | 2000 | GSTT1 | China | Asia | East Asia | population | 232 | 710 | case control | general population | age/location | RFLP | NS | NS |
| Kiyohara | 2000 | GSTT1 | Japan | Asia | East Asia | population | 86 | 88 | case control | healthy male | NO | RFLP | NS | 59 |
| Zhao | 2001 | GSTT1 | Singapore | Chinese | East Asia | hospital | 233 | 187 | case control | inpatient | age | RFLP | NS | NS |
| Hou | 2001 | GSTT1 | Sweden | Europe | Caucasian | population | 184 | 162 | case control | NA | NO | RFLP | NS | NS |
| Risch | 2001 | GSTT1 | Germany | Europe | Caucasian | hospital | 383 | 346 | case control | inpatient | NO | RFLP | NS | NS |
| Liu | 2001 | GSTT1 | USA | North America | unknown | population | 1024 | 1176 | case control | friends of cases | NO | RFLP | 54.0% | 65 |
| Sunaga | 2002 | GSTT1 | Japan | Asia | East Asia | hospital | 198 | 152 | case control | cancer free | NA | RFLP | 62.6% | 63 |
| Stucker | 2002 | GSTT1 | France | Europe | Caucasian | hospital | 251 | 268 | case control | from different disorders except cancers | age/hospital | RFLP | NS | NS |
| Lewis | 2002 | GSTT1 | UK | Europe | Caucasian | hospital | 87 | 143 | case control | patients, free of benign and malignant | NO | RFLP | NS | NS |
| Tsai | 2003 | GSTT1 | USA | North America | unknown | population | 232 | 93 | case control | healthy | NO | RFLP | NS | NS |
| Nazar-Stewart | 2003 | GSTT1 | USA | North America | Caucasian | population | 274 | 501 | case control | random digit dialing | age | RFLP | NS | NS |
| Wang | 2003 | GSTT1 | China | Asia | East Asia | population | 112 | 119 | case control | NA | age/sex | RFLP | NS | NS |
| Dialyna | 2003 | GSTT1 | Greece | Europe | Caucasian | population | 122 | 178 | case control | healthy | NA | RFLP | NS | NS |
| Cajas-Salazar | 2003 | GSTT1 | USA | North America | Caucasian | population | 110 | 119 | case control | healthy smokers | age/gender | RFLP | NS | NS |
| Ruano-Ravina | 2003 | GSTT1 | Spain | Europe | Caucasian | hospital | 125 | 187 | case control | outpatients | age | RFLP | 89.4% | 64.2 |
| Gallegos-Arreola | 2003 | GSTT1 | Mexico | America | Mexican | population | 52 | 178 | case control | healthy | NA | RFLP | NS | NS |
| Schneider | 2004 | GSTT1 | Germany | Europe | unknown | hospital | 446 | 622 | case control | outpatients | age | RFLP | NS | NS |
| Alexandrie | 2004 | GSTT1 | Sweden | Europe | Caucasian | population | 524 | 530 | case control | lab workers | NO | RFLP | 34.4% | 66 |
| Yang | 2004 | GSTT1 | USA | North America | Caucasian | population | 235 | 233 | case control | resident | NA | RFLP | NS | NS |
| Chan-Yeung | 2004 | GSTT1 | China | Asia | East Asia | population | 229 | 197 | case control | healthy | NA | RFLP | 67.2% | 53.8 |
| Lan | 2004 | GSTT1 | China | Asia | East Asia | population | 122 | 122 | case control | healthy | NA | RFLP | NS | NS |
| Belogubova | 2004 | GSTT1 | Russia | Europe | Caucasian | population | 167 | 339 | case control | health blood donors | NO | RFLP | 89.0% | 60 |
| Belogubova | 2004 | GSTT1 | Russia | Europe | Caucasian | hospital | 167 | 324 | case control | old outpatients | NO | RFLP | 89.0% | 60 |
| Sorensen | 2004 | GSTT1 | Denmark | Europe | Caucasian | population | 256 | 269 | case cohort | healthy | age/gender/smoking | RFLP | NS | NS |
| Sobti | 2004 | GSTT1 | India | Asia | East Asia | population | 100 | 76 | case control | general population | age | RFLP | 95.0% | 55.5 |
| Wang | 2004 | GSTT1 | USA | North America | Caucasian | hospital | 716 | 939 | case control | outpatients | NO | RFLP | 53.9% | 64.1 |
| Liang | 2004 | GSTT1 | China | Asia | East Asia | hospital | 152 | 152 | case control | inpatient | age/gender | RFLP | 70.4% | 60.5 |
| Cote | 2005 | GSTT1 | USA | North America | Caucasian | population | 341 | 461 | case control | random dial | age | RFLP | 53.0% | 42.4 |
| Cote | 2005 | GSTT1 | USA | North America | African American | population | 118 | 146 | case control | random dial | age | RFLP | 42.7% | 41.4 |
| Sobti | unpublished | GSTT1 | India | Asia | East Asia | population | 110 | 110 | case control | healthy | NA | RFLP | NS | NS |
| Lin | unpublished | GSTT1 | China | Asia | East Asia | population | 314 | 314 | case control | healthy | NA | RFLP | NS | NS |
| Ryberg | 1997 | I105V | Norway | Europe | Caucasian | population | 138 | 297 | case control | healthy male | NA | RFLP | NS | NS |
| Kihara | 1999 | I105V | Japan | Asia | East Asia | population | 382 | 257 | case control | healthy, smokers | NA | RFLP | NS | NS |
| Saarikoski | 1998 | I105V | Finland | Europe | Caucasian | population | 412 | 586 | case control | working-aged blood donors | NA | RFLP | NS | NS |
| Harris | 1998 | I105V | Australia | Australia | Caucasian | population | 178 | 199 | case control | general population | NA | RFLP | NS | NS |
| Jourenkova-Mironova | 1998 | I105V | France | Europe | Caucasian | hospital | 150 | 172 | case control | cancer free | NA | RFLP | 93.0% | 58.4 |
| To-Figueras | 1999 | I105V | Spain | Europe | Caucasian | population | 164 | 332 | case control | healthy | NA | RFLP | 90.6% | 59 |
| Katoh | 1999 | I105V | Japan | Asia | East Asia | hospital | 47 | 122 | case control | outpatients | NA | RFLP | 85.1% | 64.6 |
| Kiyohara | 2000 | I105V | Japan | Asia | East Asia | population | 86 | 88 | case control | healthy male | NO | RFLP | NS | NS |
| Risch | 2001 | I105V | Germany | Europe | Caucasian | hospital | 388 | 353 | case control | inpatient | NO | RFLP | NS | NS |
| To-Figueras | 2001 | I105V | Spain | Europe | Caucasian | population | 175 | 187 | case control | healthy, smokers | NA | RFLP | 91.4% | 60 |
| Stucker | 2002 | I105V | France | Europe | Caucasian | hospital | 251 | 264 | case control | from different disorders except cancers | age/hospital | RFLP | NS | NS |
| Perera | 2002 | I105V | USA | North America | Caucasian | population | 85 | 163 | case control | general population | NO | RFLP | NS | NS |
| Lewis | 2002 | I105V | UK | Europe | Caucasian | hospital | 93 | 151 | case control | patients, free of benign and malignant | NO | RFLP | NS | NS |
| Nazar-Stewart | 2003 | I105V | USA | North America | Caucasian | population | 253 | 487 | case control | random digit dialing | age | RFLP | NS | NS |
| Reszka | 2003 | I105V | Poland | Europe | Caucasian | hospital | 138 | 165 | case control | random digit dialing | age | RFLP | NS | NS |
| Wang | 2003 | I105V | USA | North America | Caucasian | population | 362 | 419 | case control | healthy | age/sex/ethnicity/smoking | RFLP | 52.4% | 60.9 |
| Miller | 2003 | I105V | USA | North America | Caucasian | population | 1042 | 1161 | case control | friends or spouse of cases | NA | RFLP | 53.0% | 66 |
| Schneider | 2004 | I105V | Germany | Europe | unknown | hospital | 446 | 622 | case control | outpatients | age | Real time PCR | 90.6% | 64.4 |
| Yang P | 2004 | I105V | USA | North America | Caucasian | population | 235 | 233 | case control | resident | NA | RFLP | NS | NS |
| Chan-Yeung | 2004 | I105V | China | Asia | East Asia | population | 229 | 197 | case control | healthy | NO | RFLP | NS | NS |
| Sorensen | 2004 | I105V | Denmark | Europe | Caucasian | population | 254 | 268 | case cohort | healthy | age/gender/smoking | RFLP | NS | NS |
| Liang | 2004 | I105V | China | Asia | East Asia | hospital | 152 | 152 | case control | inpatient | age/gender | RFLP | NS | NS |
| Cote | 2005 | I105V | USA | North America | Caucasian | population | 333 | 471 | case control | random digit dialing | age | RFLP | NS | NS |
| Cote | 2005 | I105V | USA | North America | African American | population | 118 | 146 | case control | random digit dialing | age | RFLP | NS | NS |
| Scsobti | unpublished | I105V | India | Asia | East Asia | population | 110 | 110 | case control | healthy | NA | RFLP | NS | NS |
| Harris | 1998 | A114V | Australia | Australia | Caucasian | population | 184 | 199 | case control | general population | NA | RFLP | NS | NS |
| Wang | 2003 | A114V | USA | North America | Caucasian | population | 579 | 598 | case control | healthy | age/sex/ethnicity/smoking | RFLP | 52.4% | 60.9 |
| Yang P | 2004 | A114V | USA | North America | Caucasian | population | 232 | 229 | case control | resident | NA | RFLP | NS | NS |
| Sorensen | 2004 | A114V | Denmark | Europe | Caucasian | population | 256 | 269 | case cohort | healthy | age/gender/smoking | RFLP | NS | NS |
| Saarikoski | 1998 | GSTM3 | Finland | Europe | Caucasian | population | 208 | 294 | case control | working-aged blood donors | NA | RFLP | 79.8% | 62 |
| Jourenkova-Mironova | 1998 | GSTM3 | France | Europe | Caucasian | hospital | 150 | 172 | case control | cancer free | NA | RFLP | 93.0% | NS |
| Risch | 2001 | GSTM3 | Germany | Europe | Caucasian | hospital | 389 | 350 | case control | inpatient | NO | RFLP | NS | NS |
| Tsai | 2003 | GSTM3 | USA | North America | Unknown | population | 235 | 94 | case control | healthy | NO | RFLP | NS | NS |
| Sorensen | 2004 | GSTM3 | Denmark | Europe | Caucasian | population | 256 | 269 | case cohort | healthy | age/gender/smoking | RFLP | NS | NS |
|  |  |  |  |  |  |  |  |  |  |  |  |  |  |  |
| Abbreviation: RFLP = Restriction Fragment Length Polymorphism; NA = NOT APPLICABLE; NS = not specified; MA = Mexican-American; AA = African-American | | | | | | | | | |  |  |  |  |  |
